# Minimally invasive bilateral fixed angle locking fixation of the dorsal pelvic ring: clinical proof of concept and preliminary treatment results

**DOI:** 10.1007/s00068-023-02259-z

**Published:** 2023-04-12

**Authors:** Ivan Marintschev, Gunther O. Hofmann

**Affiliations:** 1 Department of Trauma, Hand and Reconstructive Surgery, University Hospital Jena, Friedrich Schiller University Jena, Am Klinikum 1, 07747 Jena, Germany; 2 Department of Trauma and Reconstructive Surgery, Workers Compensation Hospital Bergmannstrost, Merseburger Str. 165, 06112 Halle/Saale, Germany

**Keywords:** Pelvic fracture, Posterior pelvic ring, Dorsal pelvic ring, Fragility fracture, Angular stable locking nail

## Abstract

**Purpose:**

Dorsal pelvic ring fractures may result from high energy trauma in younger patients or from osteoporosis as fragility fractures in elderly patients. To date, no strong consensus exists on the best surgical technique to treat posterior pelvic ring injuries. The aim of this study was to evaluate the surgical performance of a new implant for angle-stable fixation of the posterior pelvic ring and patient outcome.

**Methods:**

In a prospective pilot study, 27 patients (age: 39–87 years) with posterior pelvic ring fractures classified according to the AO classification (*n = *5) or to the fragility fractures of the pelvis (FFP) classification (*n = *22) were treated using the new implant. During a follow-up period of 1 year, surgical parameters of the implantation technique, complication rate, morbidity, mortality, preservation of patient mobility, and social independence were evaluated.

**Results:**

No implant misplacement or failure was observed. Two patients developed symptomatic spinal canal stenosis at L4/L5 following mobilization. MRI diagnosis proved the implant was not responsible for the symptoms. In one case, an additional plate stabilization of a pubic ramus fracture was necessary 6 months later. There was no inpatient mortality. One patient died due to her underlying oncological disease within the first 3 months. The main outcome parameters were pain, mobility, preservation of independent living and employment.

**Conclusion:**

Operative instrumentation of dorsal pelvic ring fractures should be stable enough to allow for immediate weight bearing. The new locking nail implant offers percutaneous reduction and fixation options and may decrease the generally observed rate of complications.

**Trail registration:**

German Clinical Trials Register ID: DRKS00023797, date of registration: 07.12.2020.

## Introduction

The incidence of osteoporotic sacrum insufficiency fractures (SIF) in elderly patients is rising. These injuries are associated with prolonged hospital admission, high morbidity and mortality and lead to the loss of independence in many cases. The burden on health and social care systems is tremendous.

The results of non-operative treatment are not satisfying. Surgical treatment in this patient population focusses on reduction of morbidity and mortality, improving mobility, relieving pain, and preserving social independence. Internal fixation of the osteoporotic SIF can be difficult and is not seldom followed by certain complications as screw back out, loss of reduction, fracture non-union. The development of a new implant was driven by the aim to enable a fixed angle fixation of both iliac bones with the sacrum in a percutaneous technique allowing immediate full weight-bearing mobilization of the patient.

In this pilot study, we report on our first experiences with the new implant and the operating technique as a proof of concept. All operations were completed successfully, no major complications were observed.

## Patients, materials, and methods

### Patients

In the period from August 2020 to July 2022, 162 patients with a fracture of the pelvic ring were treated at the University Hospital Jena. One hundred and twenty-two of these showed an involvement of the posterior pelvic ring. Patients with undisplaced stable fractures of the posterior pelvic ring were mobilized with weight bearing as tolerated under adequate analgesia and were not treated surgically. Those with instability, displaced fractures, and/or with persisting pain during mobilization were treated surgically employing different technical procedures.

Following CE certification of a new implant on July 6th, 2020, in a pilot study, 27 patients with instability, displaced fractures, and/or with persisting pain during mobilization have been treated with the new fixation technique.

Demographic data and reasons for in-hospital admission are shown in Table 1. All the data needed for this study were prospectively collected up to 12 months following fracture detection and surgical treatment. Informed written consent to data acquisition, study protocol, and implant use was obtained from all patients participating in this study. The study was conducted in accordance with all ethical standards of the Helsinki declaration (1983). The protocol of the study was approved by the Ethical Committee of the Friedrich Schiller University of Jena (2020-1975-MPG).

**Table 1:** Demographic Data and Reason for Admission

	*n*	*n*	*n* ∑	Years Ø	Age range	Sex	
						*f*	*m*
**Lesions without trauma**			7			7	
→ Pathological fractures in oncologic disease		2		59.0	47–71		
→ Spontaneous pain		5		76.8	60–83		
> Without osteoporosis	2						
> With osteoporosis	3						
**Trauma-associated lesions**			20				
→ High-energy trauma		5		59.4	39–79		5
> Motorcycle accident	2						
> Fall (height > 2 m)	3						
→ Low-energy trauma		15		80.2	61–87	15	
> Slipping from a chair	2						
> Stairway fall	3						
> Household fall	8						
> Fall on the street	2						
**∑**			27	74.1	39–87	22	5

Clinical data from the medical history of the patients at admission to hospital were collected prospectively and is summarized in Table 2.

**Table 2:** Medical History at Time of Admission

	*n*
→ **Musculoskeletal disorders**	
> Osteoporosis	16
> Osteoporotic vertebra fracture	4
> Spinal canal stenosis	3
> Vitamin D deficiency	4
> M. Bechterev	1
> Polyarthritis	1
→ **Metabolic disease**	
> Diabetes	7
> Gout	1
> Anemia	1
→ **Cardio-vascular disease**	
> Hypertension	8
> Peripheral arterial occlusive disease	4
> Heart insufficiency	5
> Ischemic heart disease	1
> Anticoagulation	1
→ **Neurological disease**	
> Dementia	3
> Polyneuropathy	2
> Parkinson	1
→ **Others**	
> H_2_–inhibitor	8
> Urinary tract infection	4
> Depression	3
> Chronic pain syndrome	2
> COPD	1
> Renal insufficiency	1

All patients received a CT scan at the time of first admission to the hospital. In 14 cases, additional MRI examinations were performed. Twelve patients previously had received plain radiographic imaging before admission to hospital. The five cases with high energy trauma (Table 1) were classified according to the 2018 version of the AO/OTA classification [1]. Based on CT and MRI data, the lesions of the 22 patients without or with low energy trauma (Table 1) were classified according to the fragility fractures of the pelvis (FFP) classification [2] (Table 3).

**Table 3:** Classification According to Fracture Pattern

High-energy trauma	*n = *5
AO-classification	1
61 B 2.1+ 62 B 3.2	
61 C 1.3	3
61 C 2.3	1
Low-energy or no trauma	*n = *22
FFP-classification	

Grade	Initial assessment	Updated assessment (= decision for surgery)
Unclassificable (tumor case)	1	1
I a	1	0
II b	7	2
II c	3	1
III c	1	3
IV b	9	15

Admission to in-hospital treatment and acute surgical treatment was performed in the five high energy trauma cases (time from injury to surgery 4–13 days, in one case delay of 13 days, due to pancytopenia diagnostic workup) and in the two oncologic cases.

In 12 patients (outpatient setting), a non-operative treatment approach was started in an outpatient setting and was followed by delayed surgical treatment after 2–8 weeks. Fracture progression (FFP-upgrade) associated with immobilizing pain switched the decision to surgical therapy (Table 4). Six patients (inpatient setting) without trauma or with low energy trauma were admitted for a conservative treatment regimen under pain medication WHO level III for 3–14 days (mean 8,5 days) and received a subsequent decision to surgical treatment because they remained immobilised despite analgesia (Table 4).

**Table 4:** Non-operative Treatment Attempt Followed by Surgical Treatment Decision

Outpatient		Inpatient	
* I a → IV b	2 weeks	IV b	3 days
* II b → II c	2 weeks	IV b	7 days
* II b → III c	2 weeks	IV b	7 days
* II b → IV b	2 weeks	II b	8 days
* II b → IV b	4 weeks	II b	12 days
* II b → IV b	8 weeks	IV b	14 days
II c → III c	6 weeks		
II c → IV b	2 weeks		
II c → IV b	8 weeks		
IV b →	2 weeks		
IV b →	3 weeks		
IV b →	8 weeks		

*Initial inpatient treatment

### Preoperative planning

**Fig. 1:**
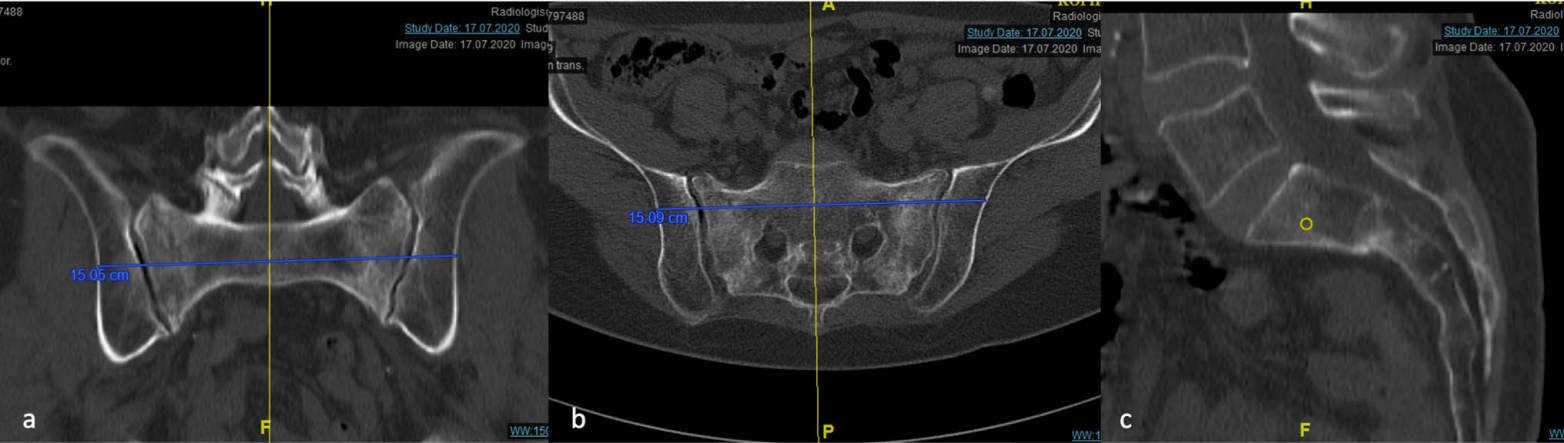
Determination of the transversal corridor of the nail: transsacral cranial view in the inlet (**a**) and the outlet (**b**) projection; determination of the optimal trajectory in the vertebral body in the lateral view (**c**)

In previous preclinical anatomic studies, the underlying principles of a dorsal interlocking pelvic ring implant have been elaborated [3]. The transversal corridor for the nail must be determined on the three standard CT levels of the S1 and/or S2 vertebral body (Fig. 1).

For a safe implantation of the nail in case of S1 sacral dysplasia, the implantation may as well be performed in the S2 vertebra. The anterior length of the interlocking ilium screws can be preoperatively estimated by calculating the corridor between the nail axis and the acetabulum.

### Description of the implant

With the SACRONAIL^®^ (SIGNUS, Alzenau, Germany), an implant for minimally-invasive stabilization of the dorsal pelvic ring is available, which offers mechanical stability in the third dimension. The implant is approved for clinical use in Europe and the USA [4, 5].

The transsacral nail (S1, S2) has a constant diameter of 8 mm and is available in lengths between 135 and 194 mm. The fixed angle of the locking screws to the nail axis is 70 degrees, and represents the anatomical corridor of the ilium axis, which was previously determined on CT-data specimens [3]. The design of the locking screws ensures a stable angular connection between the components and the cortical and cancellous bone of the posterior pelvic ring.

### Operation technique

**Fig. 2:**
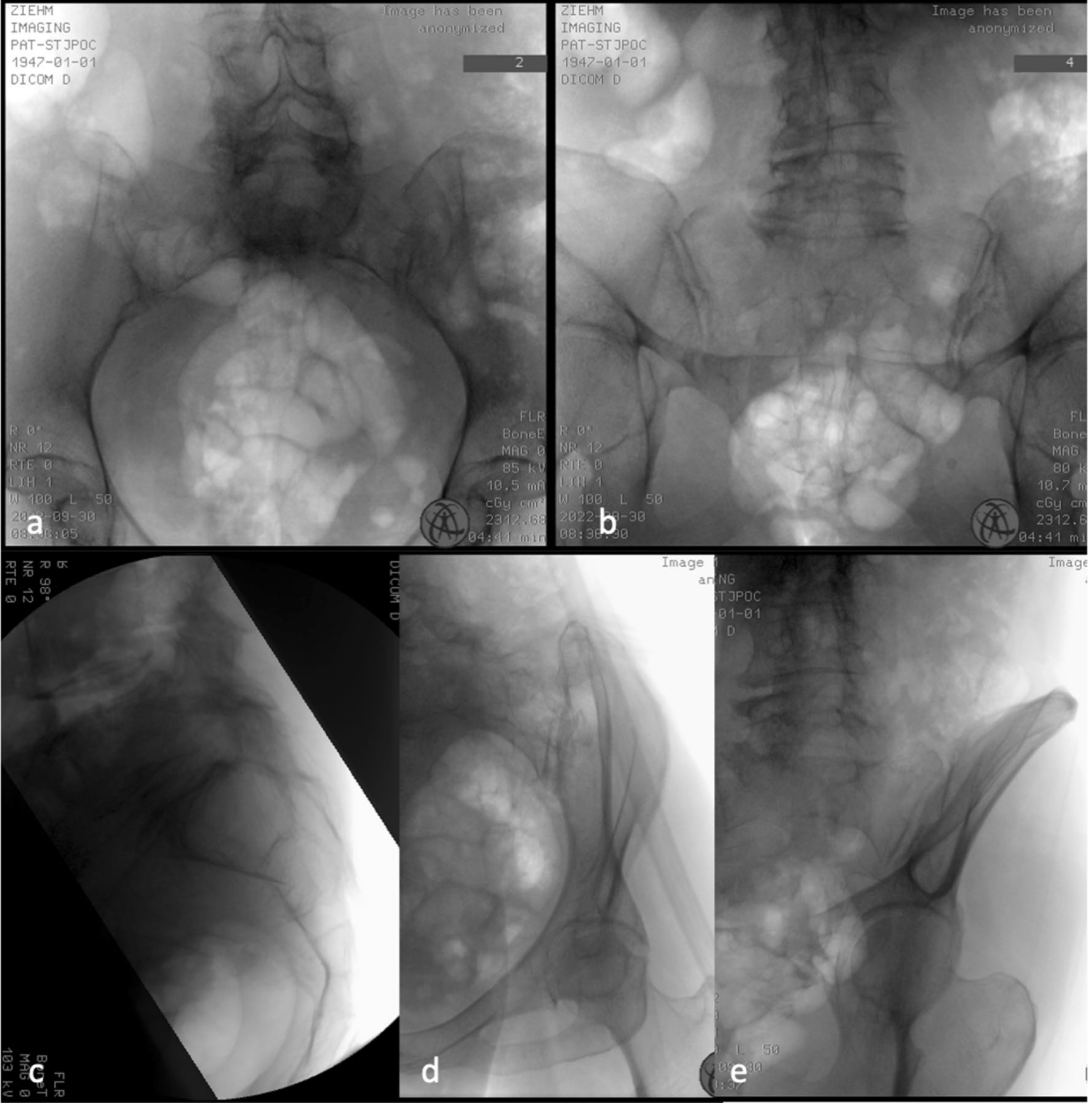
Relevant projections for intraoperative planar radiography: inlet (**a**), outlet (**b**), strict lateral (**c**); oblique view of obturator + inlet (**d**), oblique view of obturator + outlet (**e**)

The patients were placed in prone position on a radiolucent surgical table with maximum freedom of movement of the C-arm gantry for the different projections (Fig. 2).

Implantation of the nail is performed with C-arm guidance and optionally with navigation.

Fracture reduction was performed prior to implantation in a closed technique whenever possible [6]. For anatomical reduction and temporary retention, K-wires or reduction forceps may be used.

The nails were implanted either at the level of S1 or S2. With appropriate fracture morphology, it is possible to occupy both corridors.

**Fig. 3:**
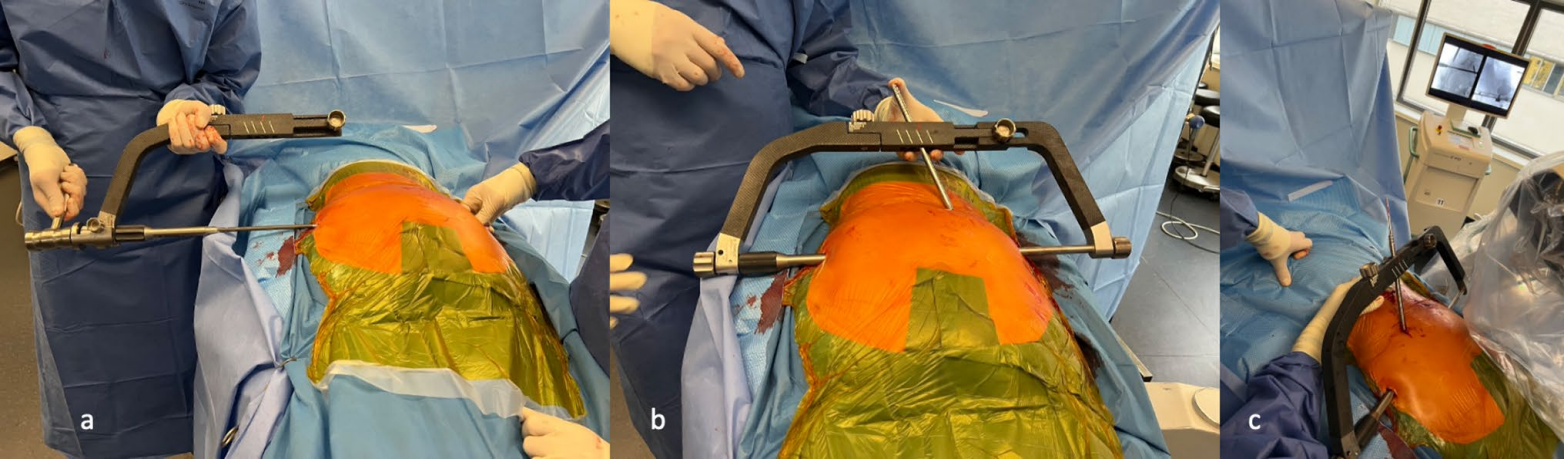
Introduction of the nail with the first half of the aiming device (**a**), aiming device fully assembled for drilling of the corridors for the interlocking screws (**b**), canulated drilling to avoid deflection of the drill on the narrow and step border of the posterior iliac crest (**c**)

**Fig. 4:**
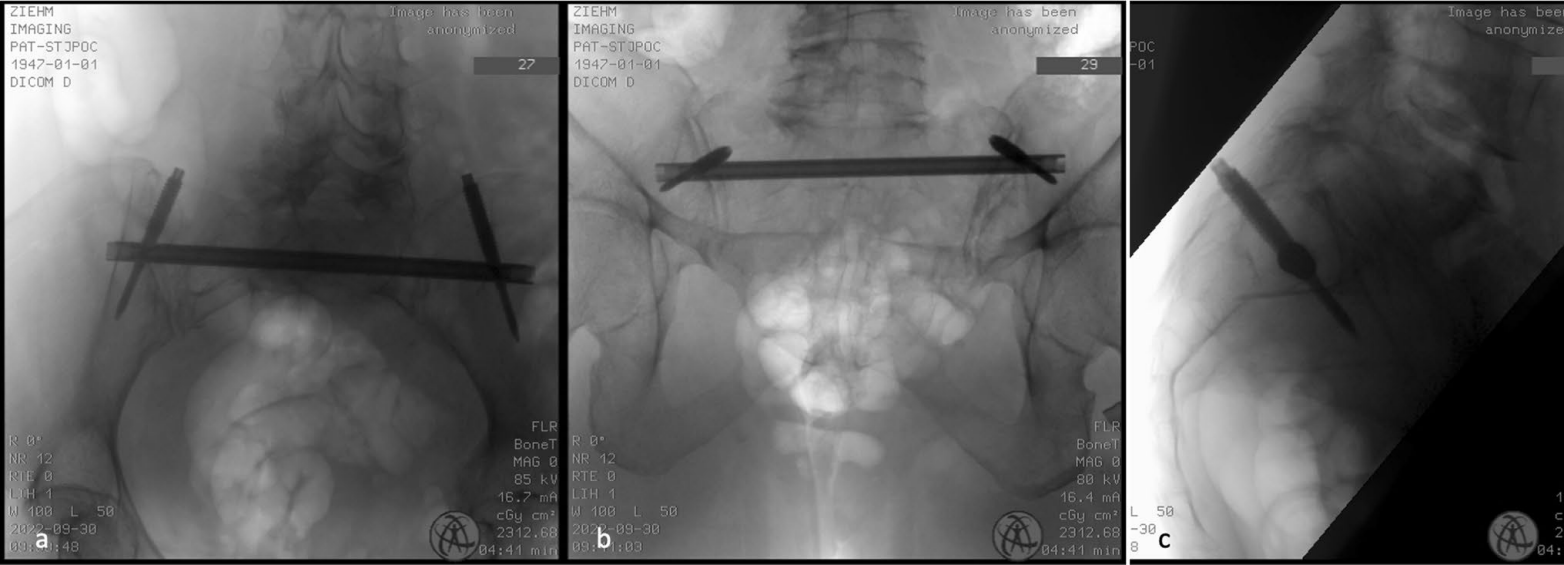
Final fluoroscopic documentation of the implanted nail in inlet (**a**), outlet (**b**) and strict lateral (**c**) projection

Under fluoroscopic guidance, a k-wire is inserted horizontally in the S1 or S2 corridor. After confirmation of correct placement, the expected nail length is measured, the corridor is reamed with a canulated drill, and the nail is assembled on the first half of a two parted aiming device. After placement of the nail, the second half of the aiming device is attached to the tip of the nail. The fixed angle interlocking screws are placed between the inner and outer border of the iliac bones after drilling over aiming sleeves (Fig. 3). A reduction and compression option through a tightening sleeve along the nail axis is available. The screws are securely connected to the nail when the torque handle has reached the maximum torque momentum as identified by a click. Final fluoroscopic check in the pelvic overview, strict lateral, inlet, and outlet projections is performed (Fig. 4).

Operative time, blood loss, and X-ray exposure were documented in all patients.

### Postoperative treatment and follow-up

All patients received prophylactic anticoagulant therapy beginning on the day of admission. One day after surgery, physiotherapy was started and patients were allowed full weight bearing as tolerated. For the evaluation of correct implant placement, all patients received a CT of the pelvis. Bone density was assessed with dual-energy absorptiometry (DXA) during the inpatient treatment. In patients with known osteoporosis, prehospital therapy was revised. In newly detected osteoporosis specific therapy was started. In-hospital and overall mortality, in-hospital morbidity, adverse events. and complications (e.g., wound healing problems, infections) were monitored.

After discharge from inpatient treatment, patients were summoned for an outpatient examination after 3 and 12 months, and in between, a telephone interview was conducted at 6 and 9 months. At the most recent follow-up examination of each patient pain and need for analgesics, mobility preservation or improvement (Table 5), social independence, and employment state (Table 6) were evaluated.

**Table 5:** Clinical Functional Outcome

Excellent	> No pain
	> Normal gait
	> Walking distance unlimited
Good	> Minor pain, occasionally
	> Analgesics only with nonsteroidal anti-inflammatory drugs
	> Walking aids (crutches, stick) occasionally
	> Walking distance ≤ 1000 m
Moderate	> Moderate pain, frequently
	> Analgesics with mild opioids
	> Walking aids (crutches, stick), continuously
	> Walking distance ≤ 100 m
Bad	> Relevant pain, continuously
	> Analgesics with strong opioids
	> Walking strongly limited (wheel chair)
	> Walking distance ≤ 10 m

**Table 6:** Residential and Employment State

Residential state	> Autonomous living at home
	> Living at home with external help
	> Residential home
	> Nursing home
Employment state	> Active in work
	> Unemployed
	> Retired from work
	> Working disability

## Results

The reported results of our first 27 patients are preliminary, and therefore only a descriptive analysis has been performed.

All operations were performed under general anesthesia. Navigation supported implantation of the nail in 19 cases. Eight patients were operated with C-arm guidance. Surgical time without navigation was 118 min, with navigation 130 min. Average X-ray exposure time was 198.5 s with navigation and 302.0 s without navigation. All the relevant data from the 27 surgical procedures are presented in Table 7.

**Table 7:** Surgery Data

	With navigation^a^		Without navigation	
	Median	Range	Median	Range
Surgical time (min)	130	71–277	118	58–449
Blood loss (ml)	20	10–200	20	10–300
X-ray exposure time (sec)	198.5	42–357	302	72–735
Dose area product (cGy / cm^2^)	1439	364–3056	2186	778–9764

	Median	Range
Nail length (mm)	160	140–182
Screw length (mm)	30/45	30/30–30/60

^a^Navigation was used in cases with additional placement of screws in the pubic rami or in cases with a narrow sacral corridor and by preference of the surgeon

The inpatient mortality rate was 0% and the all-cause mortality within 3 months of fracture was 3.7% (1 out of 27 patients, due to the underlying oncological disease). No thromboembolic events or infectious complications occurred. No wound healing problems were observed. In four cases, a postoperative urinary tract infection and one postoperative delirium were treated. Two patients developed neurological symptoms following mobilization and needed an acute diagnosis with MRI. In both cases, the implant was not responsible for the symptoms; both patients had spinal canal stenosis at the L4/L5 level. Acute surgical decompression followed, and the symptoms decreased rapidly. The average length of hospital stay was 16.3 days (range: 6–37 days).

No operative revision was necessary related to the stabilization of the posterior pelvic ring. In one case, additional surgery was required 6 months later. Persistent symptomatic non-union of a pubic ramus fracture required additional plate stabilization. No implant failure or loosening was observed. Bone healing was uneventful in all cases except one asymptomatic delayed osseous consolidation in one case of high energy trauma (male, 58 years, smoker).

**Fig. 5:**
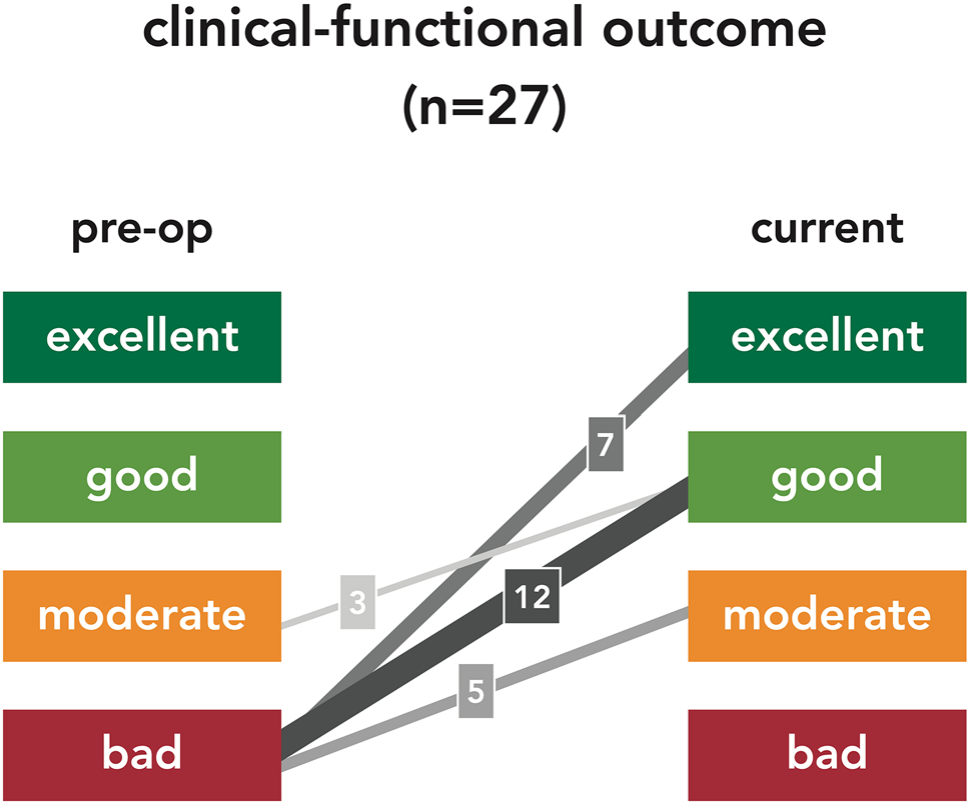
Clinical functional outcome

In all patients, an improvement of the clinical functional outcome (pain, need for analgesics, mobility, walking distance, and use of walking aids; Table 5) at the most recent follow-up was observed (Fig. 5). This demonstrates, that surgery was performed only in patients with a high level of suffering. Operative treatment contributed to a significant improvement in function and well-being.

**Fig. 6:**
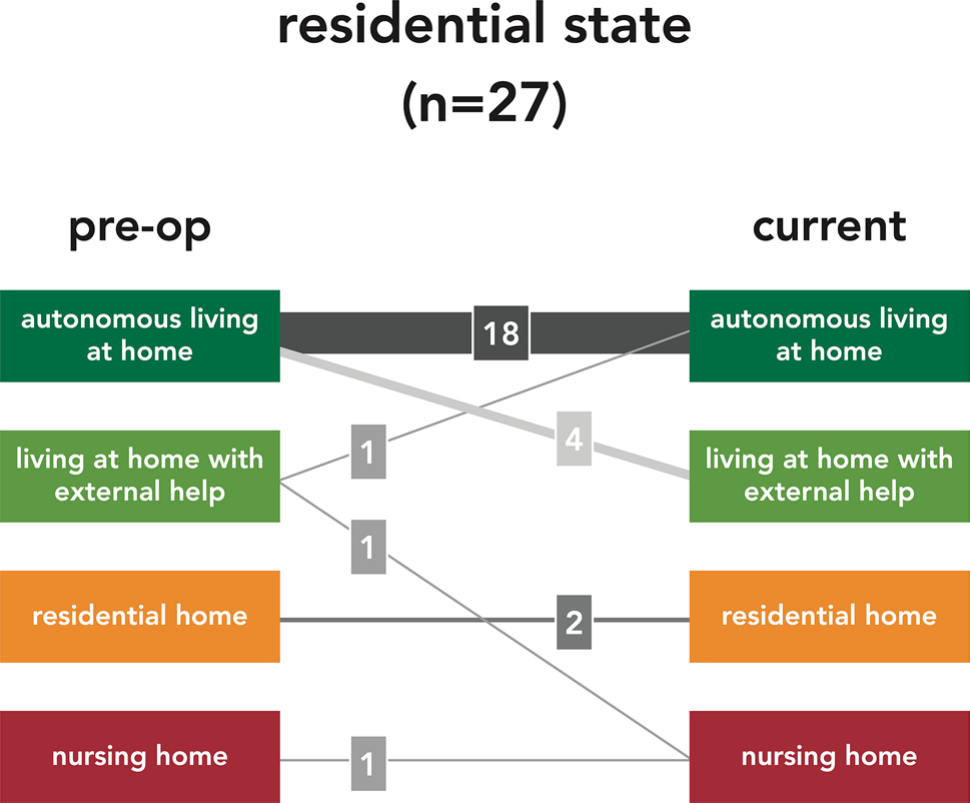
Residential state

Most patients (21 of 27) returned to their previous stage of social independence (78%); 1 patient even improved his residential state and 5 patients needed new or increased additional support (Fig. 6).

**Fig. 7:**
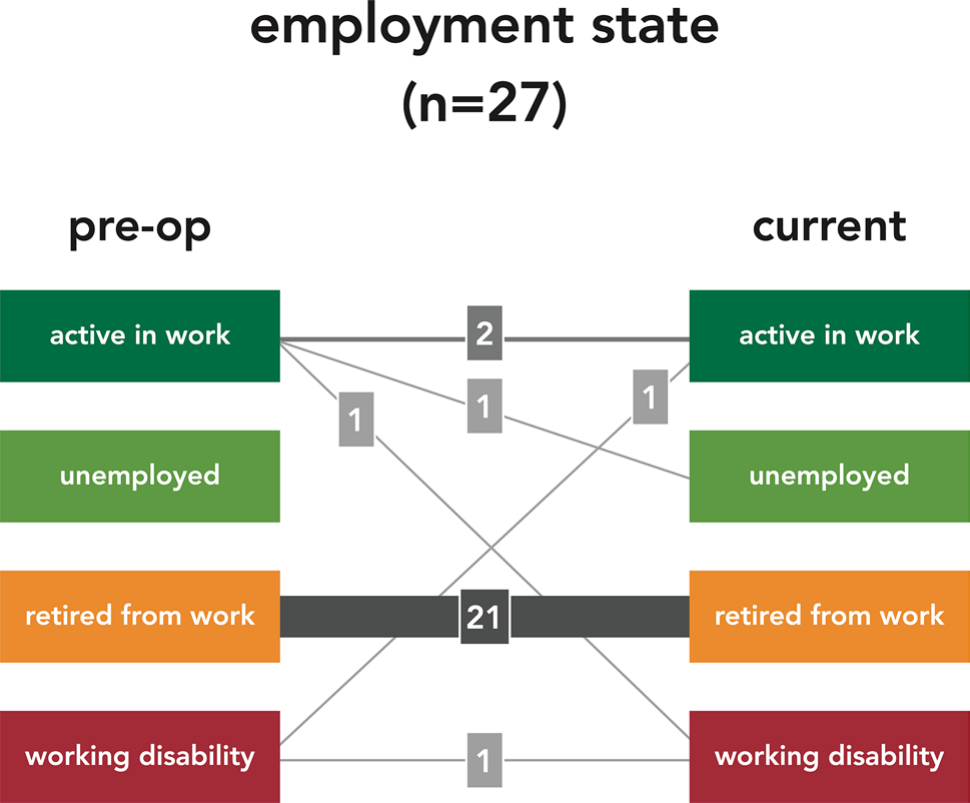
Employment state

Twenty-one patients had already retired from work before the treatment. From the four patients who were active in work prior to the fracture, two returned to work; one lost his work (unemployment). One younger patient active in work before his motorbike accident is still in rehabilitation following his injuries. One female patient even regained working ability following the operation (Fig. 7).

## Discussion

The demographic development in aging populations leads to an increased incidence of fractures in general and especially of pelvic ring fractures. For the US, Sullivan et al. found an increase in the number of osteoporotic pelvic fractures (OPF) of 24% in an analysis of an 18-year period between 1993 and 2010 [7]. Kannus et al. noted an annual increase in treated cases of 23% for Finland over 28 years (1970–1997) and predicts a tripling by 2030 [8]. In a retrospective analysis of the Dutch Hospital Database over a 26-year period (1986–2011), Nanninga et al. found a 37% increase in sacrum insufficiency fractures (SIF) and detected an association with severe comorbidity in older individuals [9]. For Germany, an incidence of SIF of 224/100,000 per year is reported [10]. These SIF not only are associated with an increase in morbidity and mortality of patients. In addition, they lead to a decrease in mobility and independence and pose a significant economic burden on health resources. For the U.S., Burge et al. calculated 873 Mio. $ total costs of OPF by 2005 and projected to grow up by 60% in 2025 [11].

Osteoporosis is the outstanding risk factor for fractures [12]. Additional risk factors include preexisting tumors and their treatment [13, 14]. Even total hip arthroplasty in the patient’s history may be a risk factor for a SIF [15]. In most cases, OPF typically are the result of inadequate low energy trauma, such as simple falls or even slipping from a chair. In quite a few cases, trauma is not remembered at all. Nevertheless, this index event can lead to a dangerous life crisis for this patient. Due to pain, their mobility is limited, and they are threatened to lose their social independence.

A non-operative treatment concept of this injury with bedrest and analgesia likely makes the osteoporosis component worse and leads to a significantly increased mortality in the short- and medium-term course and to a reduced rate of independency [16–18].

To date, no strong consensus exists on the best surgical technique to treat posterior pelvic ring injuries. Mortality following surgical treatment reaches from in-hospital mortality 0–8% [19–23] over mortality in the 1-year follow-up of 7.3–15% [19, 21, 24] and up to 50% [22] in the 2-year follow-up. Loss of independence and moving to nursing home is reported in 12–56% [21, 24].

Open reduction and internal fixation (ORIF) may be necessary in cases where closed reduction of the fracture is impossible. These procedures are complicated by high rates of wound infections [25].

In most studies, percutaneous sacroiliac screws (SIS) were used to fix the posterior pelvic ring [23, 26–31]. They permited early mobilization and provided a rapid reduction of pain levels from 3.5 to 5.7 in the VAS at hospital discharge compared with admission or immediately prior to surgery [27–33]. The procedure prevented a progression of kyphotic deformity [28, 29] and patients returned to their preinjury level of function [29]. Average hospital stay ranged from 3.0 to 23.7 days [21, 27, 30]. Most screws were inserted crossing one single sacroiliac joint (S1 or S2) [24, 27] while safe placement of the screw in the S1 vertebra can be difficult in S1 dysplasia [29]. Overall screw-related complication rate is estimated between 0 and 20% [20, 24, 27, 31, 33, 34]. The major concern is inadequate fixation in the osteoporotic bone and the risk of fixation failure [23]. Early screw loosening was observed in 1 to 8% [20, 24, 33]. Using two screws through the S1 and S2 corridor, no loosening has been observed [24, 29, 30]. Screw malposition was observed in 1 to 3.5% [20, 24, 27, 31], in double screw osteosynthesis in S1 even in 20% [20]. Collinge et al. reported subsequently insufficiency fracture of the contralateral side in 4% [23]. The reoperation rate ranged from 0 to 20% [20]. The benefit of additional sacroplasty in surgically stabilized SIF by sacroiliac screws is controversial [23, 31, 32].

Transsacral bar compression osteosynthesis (TSB) in the S1 corridor is an alternative procedure with a higher mechanical stability compared to sacroiliac screws [19, 35]. Rommens et al. recently published the results of 64 applications and reported 15.6% surgery related complications [35].

For spinopelvic stabilization (SPS), biomechanical advantages compared to SIS and TSB are reported [36, 37] resulting in significantly lower incidences of implant dislocation [38]. Implant failure and implant loosening rates were reported in 5 to 30% [19, 39]. Misplacements of pedicle screws are observed in 4% [40]. Severe skin complications and wound infections with need for operative revision range between 4 and 50% [40–44]. Patients with a combination of procedures (e.g., SPS and TSB) showed significantly better stability of the osteosynthesis and a better rehabilitation outcome [6, 19, 22, 40, 45].

As the result of an analysis of the data from the German Pelvic Trauma Registry (5665 patients over a 22-year observation period), Rollmann et al. concluded that the predictive demographic change and a shift toward more severe injury patterns in the elderly population is a challenge needing development of new surgical concepts for geriatric patients with SIF [46]. A bilateral, angle-stable implant for the dorsal pelvic ring offers a further advantage from a pathophysiological and biomechanical point of view. Bilateral SIF can develop from a unilateral vertical lesion. At the first moment following monolateral SIF, the overlying sacral ligaments are intact. With time, a contralateral lesion may occur and result in progressive bilateral vertical instability [47]. Sacroiliac screws (SIS) or transsacral bars (TSB) as minimally-invasive procedures cannot guarantee stability in three dimensions of the pelvis, even in the case of two corridor (S1 and S2) implantation.

Preliminary results are promising and give confidence that the implant can enable a stable fixation of the posterior pelvic ring with immediate full weight bearing. No wound healing or infectious complications were observed up to now. The two neurological adverse events following surgery and postoperative mobilization were not associated with malreduction or implant misplacement. Despite immediate mobilization following surgery in all 27 patients, we experienced no fracture reduction loss or loosening of the implants [19, 20, 24, 27, 31, 33, 39]. The study emphasizes the impact of early full weight-bearing mobilization to avoid inpatient mortality [16, 18–23], to reduce complication rates [17, 20, 21, 23, 24, 31, 33–35, 40–44], and to reduce overall mortality [16–19, 21, 22, 24]. The most apparent impact of the study was observation of a low risk level state of social independence loss [16–18, 24] and a high level of preservation of employment.

There are several limitations to this study. First, the validity of this study is limited by the volume of the study population and the age and injury mechanism heterogeneity of the case series. Second, compared to validated anatomical scores or PROMs, a simple functional-outcome evaluation was preferred in this preliminary surveillance of the surgical technique and the implant safety and reliability. The advantages and limitations of the new implant compared to standard operative procedures must be critically evaluated in further randomized prospective studies.

## Data Availability

Raw data of the study are not publicly available as the study participants have not given a signed consent for public insight and use and their privacy is respected under the European General Data Protection Regulation. Anonymized raw data are available on request.
